# Sexually transmitted infections on the border between Brazil and French Guiana

**DOI:** 10.3389/fpubh.2023.1059137

**Published:** 2023-01-25

**Authors:** Mathieu Nacher, Flavia Divino, Cyril Leborgne, Valmir Correa, Sébastien Rabier, Aude Lucarelli, Sophie Rhodes, Mélanie Gaillet, Dorinaldo Malafaia, Cyril Rousseau, Alice Sanna, Margarete Gomes, Antoine Adenis, Paulo Peiter, Céline Michaud

**Affiliations:** ^1^INSERM CIC1424 Centre d'Investigation Clinique Antilles Guyane, Cayenne, French Guiana; ^2^Centres délocalisés de prévention et de soins, Centre hospitalier de Cayenne, Cayenne, French Guiana; ^3^Laboratorio De Fronteira De Oiapoque, Oiapoque, Brazil; ^4^COREVIH Guyane, Centre hospitalier de Cayenne, Cayenne, French Guiana; ^5^IDSanté, Saint Georges de l'Oyapock, French Guiana; ^6^Superintendência de Vigilância em Saúde, Macapa, Brazil; ^7^Oswaldo Cruz Foundation (Fiocruz), Rio de Janeiro, Brazil

**Keywords:** border, Brazil, French Guiana, HIV, sexually transmitted infections, testing, cooperation

## Abstract

**Purpose:**

The border between the State of Amapa, Brazil, and French Guiana is mostly primary forest. In the Oyapock basin, socioeconomic circumstances have fueled sex work, gold mining and the circulation of sexually transmitted infections. Given the lack of comprehensive data on this border area, we describe the different sexually transmitted infections along the Brazil/French Guiana border and the testing and care activity.

**Methods:**

We conducted a review of the available scientific and technical literature on sexually transmitted infections in this complex border area. Temporal trends were graphed and for Human Immunodeficiency Virus (HIV) we estimated incidence using the European Center for prevention and Disease Control modeling tool.

**Results:**

Until 2019, 26 of the 46 HIV-infected patients followed and treated in Saint Georges de l'Oyapock were residing on the Brazilian side in Oiapoque. Virological suppression was only achieved for 75% of treated patients; but dropped to 62% during the COVID-19 epidemic. In 2019, cooperation efforts allowed HIV care in Oiapoque, resulting in the transfer of Brazilian patients previously followed on the French side and a substantial increase in the number of patients followed in Oiapoque. The average yearly HIV serological testing activity at the health center in Saint Georges was 16 tests per 100 inhabitants per year; in Camopi it was 12.2 per 100 inhabitants. Modeling estimated the number of persons living with HIV around 170 persons, corresponding to a prevalence of 0.54% and about 40 undiagnosed infections. The model also suggested that there were about 12 new infections per year in Saint Georges and Oiapoque, representing an HIV incidence rate of 3.8 cases per 10,000 per year. HPV prevalence in Saint Georges ranges between 25 and 30% and between 35 and 40% in Camopi. Testing activity for other sexually transmitted infections markedly increased in the past 5 years; the introduction of PCR for chlamydiasis and gonorrhea also had a substantial impact on the number of diagnoses.

**Conclusions:**

The ongoing cooperation between multiple partners on both sides of the border has led to remarkable progress in primary prevention, in testing efforts, in treatment and retention on both sides of the border. In a region with intense health professional turnover, nurturing cooperation and providing accurate assessments of the burden of sexually transmitted infections is essential to tackle a problem that is shared on both sides of the border.

## Introduction

Border areas are frequently hubs of complex social and economic exchanges. Borders also constrain human movements, and, through this, diseases (1). The epidemiology, prevention and care of HIV and other sexually transmitted diseases are also influenced by borders (2, 3). Socioeconomic inequalities between countries may enhance transactional sex and sex work; furthermore, the decrease of social scrutiny one's countrymen on the other side of the border may facilitate casual sex. Such dynamics have led to emerging HIV epidemics in cities along the US-Mexico border (4). Differences in the level of health care facilities, or stigma, may also lead some patients to seek care on the other side of the border.

The global prevalence of HIV has exceeded 1% for over 3 decades in the different territories of the Guiana Shield, an ancient geological formation that includes Eastern Colombia, Eastern Venezuela, Guyana, Suriname, French Guiana, and the northern Brazilian states, notably Amapa, which borders French Guiana. The per capita health expenditure in 2018 was 1,498 US dollars (612$ from government, 839$ from voluntary insurance) in Brazil vs. 2,500 US dollars in French Guiana (5, 6). The border between Brazil and French Guiana is 730 km long, and it is mostly unenforced, apart from Oiapoque municipality (28,534 inhabitants) and Saint Georges de l'Oyapock (4,277 inhabitants) (7, 8). The rest of the border is mostly surrounded by primary Amazonian forest and the remaining villages can only be reached by canoe or small aircrafts. The population density in Oiapoque municipality (which covers the Eastern border with French Guiana) is 1.26 per km^2^;that the population density of Laranjal do Jari is 1.69 per km^2^, but most live far from the border with French Guiana, whose Southern border is thus is devoid of official inhabitants. The total population of Eastern French Guiana has tripled in the past 50 years and has stabilized around circa 6,900 persons, representing a global density of 0.5 person per km^2^, the highest density being found in the commune of Saint Georges de l'Oyapock (1.8 per km^2^), which lies across the town of Oiapoque, which is also the most populated area along the border. Hence, the “population hotspot” and the “hub” on the border between French Guiana (it is part of France and the European Union) and Brazil only entails an estimated total of 35,000 persons. On the French Guiana side, the population is young (62% are aged < 30 years) and mostly composed of creoles and Amerindian tribes. On the Brazilian side, the population is also young (64% aged < 30 years) and mostly of Amerindian descent. A particular population of interest, which moves back and forth between Brazil and French Guiana, is represented by gold miners “garimpeiros,” a population approaching 10,000 (9) working in scattered illegal mining sites in the forests of French Guiana. A large proportion of garimpeiros enter French Guiana through the Oyapock border and often fall back to Oiapoque, which hence serves as a base station and logistical support hub for gold miners (10).

**Figure 1:**
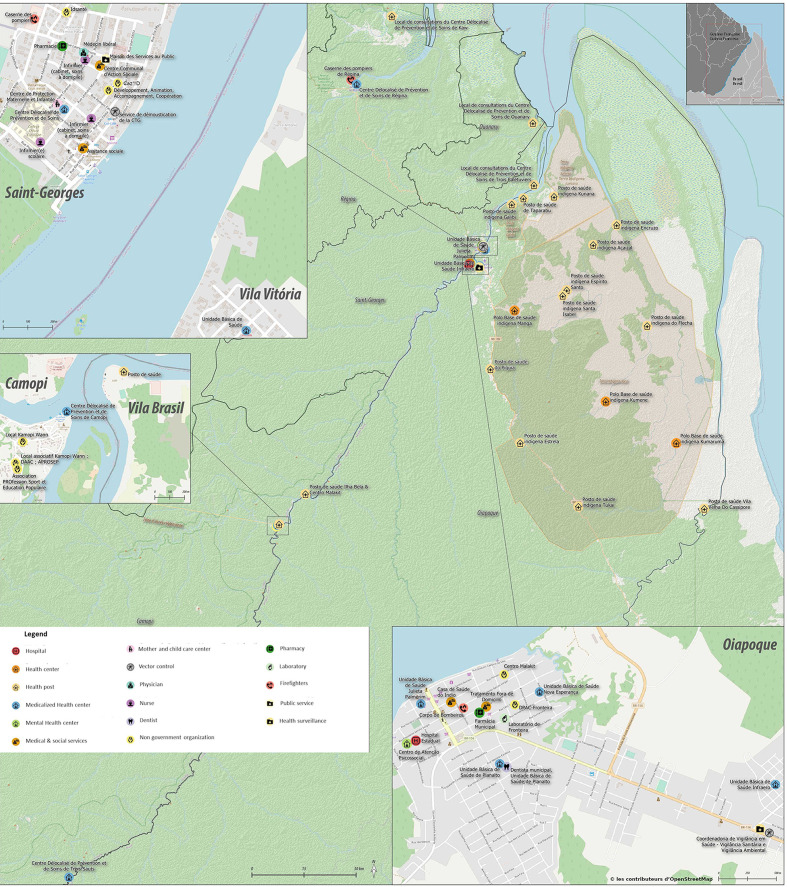
Map of the main border towns on the Oiapoque river between French Guiana and Brazil.

Given the vastness of the territory traversed by the border river, its administrative boundary function is hence often virtual, and relatives and friends are often scattered between two countries. This transborder living space leads to what is called pendular migration with people moving back and forth between sides (11). Given these continuous population movements, it is key to act on both sides of the border and to coordinate the respective health professionals and NGOs (12). Each country has pressing national plans to implement, central authorities to report to, resulting in two distinct and poorly connected networks that are driven by their own internal constraints. However, health professionals face the same diseases in populations that are similar and interact more often than administrations actually do; it is thus crucial to coordinate epidemiologic surveillance, prevention, and care on both sides of the border (12). Figure 1 maps the main health structures on the border. Epidemiologic knowledge about trends on both sides of the border is also crucial for a shared diagnosis and prioritization of concerted public health interventions; it is important because health problems have local specificities and population representations may also be quite different from other areas. Hence, while the overall HIV prevalence in Brazil is estimated at 0.6%, the prevalence in Northern states is much higher and increasing (13). In French Guiana, the overall prevalence is estimated to be between 1.18 and 1.35% (14). However, because of the singular context of this border area, such general prevalences may be substantially different from reality. Given the epidemic progression in Northern Brazil, Federal, State, and Municipal levels in Brazil raised the alarm over a decade ago and organized workshops with the Health Regional Agency of French Guiana and multiple stakeholders (15). This led to regular interactions, yearly joint events, and a better acquaintance of those working on both sides. Since then, the Regional Cooperation for the fight against HIV recruited a French-Brazilian physician to coordinate cooperation on a border where health professional turnover is intense and makes mutual knowledge challenging. European funding with Interreg Caraïbes also briefly contributed, and since 2017, the PCIA (Programme Cooperation Interreg Amazonie) funds a specific programme, OCS (Oyapock Cooperation Santé) uniting Cayenne hospital's health centers, IDSanté, DAAC, DPAC fronteira, a programme that has accentuated the focus on sexual health at the border (16). On the Brazilian side, several university theses studied –mostly qualitatively– the situation in Oiapoque (17–22). Hence, on a territory with a context that is conducive to STI transmission, where health professionals and non-government organizations are mobilized to act, there has never been a thorough analysis of the available fragmented information on STIs and the public health response at the scale of the Border.

Our objective in this context was thus to provide a synthetic narrative review of the available evidence on STIs and efforts to tackle them on the border between French Guiana and Brazil.

## Methods

### Published information sources

Scientific publications, technical reports, and MD, PhD, and Master's theses in English, French and Portuguese dealing with HIV and other STIs on the border region were gathered to analyze the situation from both sides of the Border (Supplementary Figure S1). Quantitative and qualitative studies were included as long as they related to Oiapoque, Saint Georges de l'Oyapock, Camopi, and Vila Brasil, a hamlet across from Camopi. Health workers, researchers and non-government organizations from both sides of the border (the authors of the present article) then reviewed this trove of data to get a better grasp of the situation along the border.

### Data sources

Databases from the HIV programme of French Guiana (COREVIH) and from the Centres délocalisés de Prévention et de Soins of Saint Georges de l'Oyapock and Camopi (Health centers in remote villages in French Guiana) were used to provide information on testing, and care and treatment activities. Testing activity and number of cases per year were plotted for different pathogens using excel. The aim of the study being descriptive, no hypothesis testing or multivariate analyses were conducted. Denominators were obtained using census data from both sides.

### Diagnostic methods

HIV diagnoses relied on positive Elisa and/or INSTI rapid tests, confirmed by Western Blot. For Syphilis, diagnosis was based on the presence of a positive TPHA and a positive VDRL test in the patient's serum. For chronic hepatitis B, the presence of HBS antigen in the serum was the criterion used. For *Chlamydia trachomatis* and *Neisseria gonorrhea*, polymerase chain reaction was used to amplify each pathogen's specific sequences from urine or vaginal samples. On the French side all analyzes were conducted by the hospital laboratory. On the Brazilian side analyses were performed in different structures in Oiapoque, including HIV Western blot confirmation and viral load.

### Modeling the HIV epidemic

The ECDC HIV Modeling Tool has been developed by European Center for Disease Prevention and Control to provide estimates of the number of people living with HIV, including those that have not yet been diagnosed. It can also estimate the yearly number of new HIV infections. To do so, it only needs HIV surveillance data. To estimate the number of HIV persons in the Oyapock/Saint Georges population basin from the available data, we worked with the assumption that the HIV epidemic in Saint Georges de l'Oyapock and its neighboring Oiapoque was a single phenomenon; we pooled the number of new diagnoses and assumed that a third of new diagnoses were AIDS cases and that new AIDS cases within a year were 40% greater than that (assumptions that mirrored the observed patterns in the Cohort of French Guiana), we modeled the epidemic using the ECDC HIV modeling tool using the incidence method with 1,000 bootstrap iterations (23).

## Results of the review of available information

### HIV/AIDS

The incidence of AIDS in French Guiana was 11.8 per 100,000; in Amapa state it was 18.7 per 100,000 (15). In Brazil, there is a trend across states showing substantially higher incidences in State capital cities than in the rest of the State (15). Amapa was the Brazilian state with the greatest increase of HIV/AIDS mortality between 2010 and 2020 (+240%), while other non-north states had a decline in HIV/AIDS mortality. Between 2007 and 2021, 1,801 cases of HIV were notified in Amapa, with a recent increase since the mandatory reporting of HIV in 2015 (15). Supplementary Figure S2 shows that Oiapoque had the state's second highest number of HIV cases per 100,000. During the same period, 598 pregnant women with HIV were also notified in Amapa. Amapa also had the second highest incidence of pediatric HIV in Brazil (15). After 2015, the SINAN reported around 250 new HIV diagnoses per year (15), which represents an incidence of 295 per million inhabitants; by contrast, in French Guiana, the incidence of new diagnoses in 2018 was 896 per million, a much higher incidence than Amapa that may, in part, reflect the exhaustiveness of data reporting (24). Overall, while HIV is preoccupying in Amapa, the HIV epidemic situation in Oiapoque is among the worst, with new infections nearing the levels observed in French Guiana–between 50 and 70 per 100,000 inhabitants.

On the French side of the border, the 2 health centers administratively attached to Cayenne general hospital offer primary care and may perform free biological testing (sending samples to Cayenne) and HIV care. Pre and post exposure prophylaxis are also available in Saint Georges de l'Oyapock. Emergencies or specialized consultations are referred to Cayenne by road (Saint Georges) or helicopter (Camopi). Non-government organizations are well-established in Saint Georges and collaborate closely with the health center; IDSanté provides sexual and reproductive health education and provides condoms; DAAC (Developpement, Animation, Accompagnement, Cooperation) organizes prevention events, also provides condoms, and employs health mediators to accompany HIV patients for their follow-up; the red cross and Médecins du Monde sometimes conduct mobile HIV-testing missions. On the Brazilian side, there is a hospital and a primary care clinic (UBS) that provided some unknown quantity of HIV-testing and condom distribution. The non-government association DPAC is a twin NGO with DAAC in Saint Georges, which provides precious coordination around sexual health efforts and around individual patients moving back and forth between both sides of the border.

In this area of high population mobility, 39 known persons living with HIV were lost to follow-up from Saint Georges health center, and 4 patients died in the past decade. Among patients entered in the HIV cohort database, a third had CD4 counts < 200 per mm^3^ at the time of diagnosis and 14/66 (21.1%) were at the AIDS stage with on average 1 new AIDS case per year for a village of 4,188 persons.

**Figure 2:**
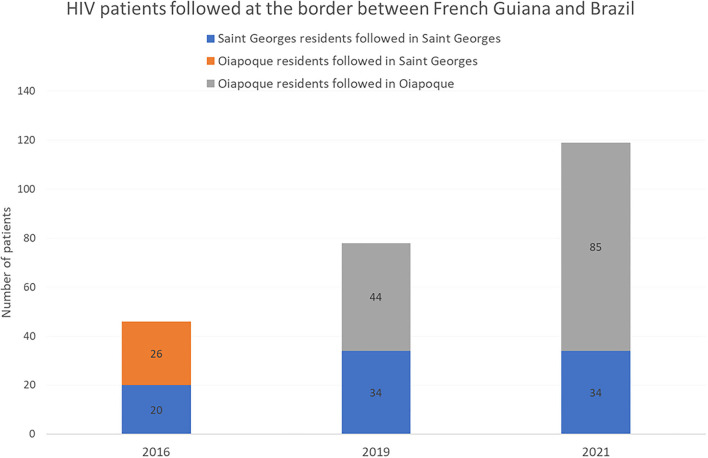
Evolution of the number of persons living with HIV followed in Saint Georges de l'Oyapock and Oiapoque before and after the availability of HIV care in Oiapoque.

Most HIV patients living in Oiapoque were followed and treated until 2019, an unknown number were treated in Macapa every 3 months (12 h by bus, in the dry season). Until 2019, 26 of the 46 HIV-infected patients followed and treated in Saint Georges de l'Oyapock were residing on the Brazilian side in Oiapoque. The illegal status and boat fees were obvious barriers to care, and virological suppression was only achieved for 75% of treated patients; it is of note that in Saint Georges virological suppression dropped to 62% during the COVID-19 epidemic. The alternative was the long bus ride to Macapa, and the necessity to pay transport and accommodation for each visit, conditions that constitute substantial barriers for patients. After prolonged efforts to implement treatment delivery and to ensure biological follow-up, cooperation efforts finally succeeded, in 2019, to offer HIV care in Oiapoque; this resulted in the transfer of Brazilian patients previously followed on the French side, and in a substantial increase of the number of patients followed in Oiapoque (Figure 2 and Supplementary Figure S3). There are 3 complementary hypotheses to explain this increase in patient numbers: first, we are perhaps uncovering an active epidemic in an area where large numbers of sex workers, mobile gold miners, and even frequent sex tourism are compounded by insufficient testing and substantial barriers to access treatment, and this combination may fuel an epidemic that is not under control; however, a second and less bleak hypothesis would be that, given the efforts in the past decade, HIV testing has greatly improved and succeeded in doing what it should do: identify infected persons so they can get treated; Finally, another optimistic but highly plausible hypothesis is that the offer of treatment services in Oiapoque is attracting patients that were previously untreated and the increase in patient numbers is a sign of its success. These 3 hypotheses are not mutually exclusive. However, given the recent history of reinforced testing, preexposure prophylaxis, coordination and efforts around sexual health on the border would be expected to have favorable impacts despite the social context and vulnerable groups present on the border.

**Figure 3:**
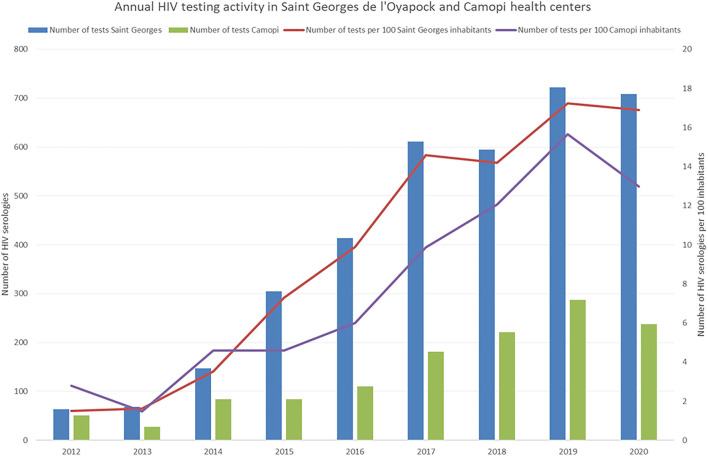
Evolution of the HIV testing activity on the French side of the border.

The bulk of HIV on the border is mostly divided between Oiapoque and Saint Georges, Camopi a small remote town 6 h upstream on the Oyapock river has < 5 cases. The average yearly HIV serological testing activity at the health center in Saint Georges was 16 tests per 100 inhabitants per year; in Camopi it was 12.2 per 100 inhabitants with a marked increase in the past 5 years (Figure 3). These testing figures are lower than the overall testing activity in French Guiana (20.3 per 100) but higher than testing activity in mainland France (fluctuating around 10 per 100). On the Brazilian side, rapid tests are used to diagnose HIV but there is no information on the number of HIV tests per year per 100 inhabitants.

We have shown that in French Guiana Bcar lineages are predominant over B pandemic lineages (25, 26). In Brazil, B pandemic lineages (and to a far lesser degree F) largely predominate; the 3 Brazilian states that have the greatest proportion of Bcar lineages are Maranhao, Roraima, and Amazonas (27), areas that are notoriously linked to gold mining and where many garimpeiros come from, notably, for French Guiana, Maranhao. This suggests that, through migration to goldmines on the Guiana Shield and through the local networks of sex workers, garimpeiros may be a population bridging the Brazilian epidemic and the Caribbean epidemic.

**Figure 4:**
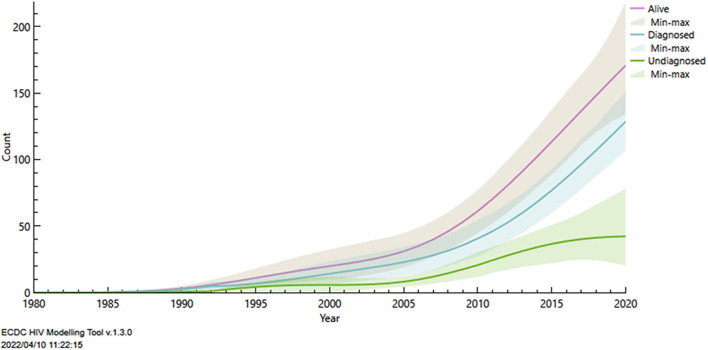
Modelling of the HIV epidemic in the Saint Georges/Oiapoque basin using the ECDC modelling tool in order to estimate the total number of persons living with HIV.

The absence of distinction between HIV and AIDS in Oiapoque data was a limitation that required us to make educated guesses about AIDS cases. Furthermore, given that new notified HIV infections are aggregated data, we had no way to verify that some cases were counted twice (in Saint Georges and Oiapoque), which suggests that our modeling scenario may yield somewhat higher estimates than the reality. Despite its limitations, the present ECDC model, shown in Figure 4, provides estimates of the number of persons living with HIV (about 170 corresponding to a prevalence of 0.54%) and of the number of undiagnosed infections (about 40). Prior attempts to do so first postulated that prevalence in the area was 1% (it may not be that at all) and computed the number of persons living with HIV supposed to be in the area. This 1% working hypothesis seems more arbitrary and fragile than our modeling attempt, which still follows the dynamics of actual number of cases. The model also suggests that there are about 12 new infections per year in Saint Georges and Oiapoque (Supplementary Figure S4), given the population basin this would represent an HIV incidence rate of 3.8 cases per 10,000 per year.

### Human papillomavirus infections

In a recent study, we observed that HPV prevalence in Saint Georges ranged between 25 and 30% and that in Camopi it ranged between 35 and 40% prevalence, a majority of infections were by high risk genotypes (28, 29). Cervical cancer in French Guiana is the second most frequent cancer among women 24 per 100 000 women with a greater incidence along the Oyapock and Maroni rivers. On the Oyapock, the standardized incidence was >31 per 100,000 woman-years and in Camopi it was 113 per 100,000 woman-years (30). This high incidence of cervical cancer has been hypothesized to result from: early sexuality, multiple partnerships, screening and access to care delays, immunogenetic susceptibility to HPV viruses, or particular oncogenic variants. The 3 most frequent HPV genotypes on the Border area were 52, 16, and 31. E6 and E7 oncogen sequence diversity analysis showed that, for most genotypes, there was a strong ethnic clustering suggesting that notably that Amerindians in Camopi have highly clustered virus genotypes (31). This possibly reflected different ancestral virus populations and endogamy with rare sexual contacts with other ethnic groups in Camopi, which is located in a protected area where travel requires prefectoral permits.

The non-avalent HPV vaccine covers most circulating genotypes on the Oyapock but vaccine coverage is so far lower in French Guiana (14.1%) than in Mainland France (23.7%), and on the Oyapock, where HPV is so prevalent, vaccination rates remain close to zero. In French Guiana HPV testing strategies that could be game changers in these remote areas are now authorized and reimbursed. However, WHO's aim of 90 × 70 × 90 by 2030 (90% of girls vaccinated before 15 years old; 70% of women screened by an HPV test twice in their lifetime; 90% of screen-positive women treated) (32) seems far beyond our reach. This is presumably the case on both sides of the French Guiana-Brazil border, and should definitely be prioritized if we are to reach the target. However, if we consider the population basin on the border, this does not include so many women and, despite logistical challenges due to distance, 90 × 70 × 90 by 2030 seems attainable if both countries have the will to do it.

### Hepatitis and other sexually transmitted infections

Hepatitis serological screening is far more frequent than bacterial sexually transmitted infections, which remains mostly syndromic, thus presumably overseeing a substantial proportion of persons that are actually infected. Given the absence of data on Sexually transmitted infection diagnoses in Oiapoque, it was not possible to estimate results for the Oiapoque-Saint Georges basin.

### Hepatitis B

Supplementary Figure S5 shows HBV testing activity more than tripled in Saint Georges. Overall, the proportion of active HBV infections among those tested was 1.2% in Saint Georges and 0.6% in Camopi. At Saint Georges health center between 2017 and 2021 there were 32 patients followed for chronic hepatitis B (10 women and 22 men). This figure is unknown for Oiapoque but a study on garimpeiros working in French Guiana found a prevalence above 4% among men and women for HBS antigen positivity (33).

### Chlamydia

Between 2017 and 2021, there were 24 cases of chlamydiasis [18 women (6 Brazilian) and 6 men (1 Brazilian)]. The recent introduction of PCR has led to a substantial increase in screening (Supplementary Figure S6). Even when using the population of Saint Georges aged 15 or more as denominator (60% of the total population), this incidence is far lower than estimates in the Americas where chlamydiasis incidence estimates exceed 60 per 1,000 adult men and women (34).

### Gonorrhea

Between 2017 and 2021, there were 24 cases of gonorrhea [10 women (4 Brazilians) and 14 men (5 Brazilians)]. As for *Chlamydia*, the introduction of PCR has substantially increased screening activity (Supplementary Figure S6); it is likely that its non-invasive nature and sensitivity lead to transitioning from diagnosis when facing syndromes to broader screening of persons who do not necessarily have symptoms. Given the COVID-19 pandemic wave on the border between Brazil and French Guiana, overall testing activity declined in 2021. However, there again, the observed annual incidence among those aged 15 years or more in Saint Georges is far lower than estimates in the Americas at 20 per 1,000 adult men and women (34). These crude comparisons should be considered with caution because screening efforts and tools may widely differ as methods to calculate incidence and because we have no data from the Brazilian side. Nevertheless, the lower incidences of chlamydiasis and gonorrhea seems to counter the common belief that they are widespread in this region. This may result from the considerable prevention and testing efforts in the region in the past decade.

### Syphilis

Syphilis testing activity substantially increased on the French side (Supplementary Figure S7). Between 2017 and 2021, there were 34 cases of syphilis [17 women (13 Brazilians) and 17 men (9 Brazilians)]. The observed annual incidence among those aged 15 years or more in Saint Georges was thus 2.8 per 1,000, which is greater than the 1.7 per 1,000 estimates in the Americas and contrasts with the lower incidences of Chlamydiasis and gonorrhea relative to the Americas (34). It is of note that the proportion of Brazilian patients among syphilis cases was over 3 times greater than those who had gonorrhea and/or chlamydiasis, and this was statistically significant. Perhaps, systematic screening for syphilis during pregnancy on the French side allows to identify and treat women and their partners, and thus decreases the prevalence of active infections; this would imply that screening is less systematic in Brazil hence resulting in a greater circulation of syphilis. A study on garimpeiros working in French Guiana found a prevalence of positive treponemic tests of 8.9% among men and 17.9% among women (31).

## Discussion

The Brazil-French Guiana border is a place of economic, cultural, social and sexual exchanges. The educational level is often low, and poverty is widespread (35, 36). Drug use –notably cocaine and crack cocaine—is common. The area is also a supply and transit hub for illegal gold mining. Sex work is widespread with multiple venues offering sexual services on the Brazilian side, customers may be miners or persons coming from French Guiana. A study showed that HIV prevalence among gold miners at resting sites across from French Guiana was 1.4% (37). Sex work is indeed closely related to gold mining and many sex workers leave to work for a few months in isolated gold mines. A survey of 213 sex workers (35 in Saint Georges and 178 in Oiapoque) conducted in 2012 showed that 31% of them had never performed an HIV test. For those included in the survey (it was not exhaustive), this represented nearly 9 sex workers per 1,000 inhabitants in Saint Georges and 8 sex workers per 1,000 inhabitants in Oiapoque. Both of these low estimates are more than triple the numbers in Thailand (2 per 1,000), 15 times estimates in France (0.5 per 1,000), and more than double the estimates from Brazil 2.6 per 1,000) thus underscoring the magnitude of sex work in the area. Overall, 7.6 % of sex workers had sex without a condom. Twenty-six percent of sex workers did not know where to get an HIV test. About 58% of sex workers had worked at a gold mining site, (65% for sex workers in Oiapoque). Three quarters of sex workers did not live in Oiapoque permanently and were only in transit there. Those who lived in Oiapoque were less likely to have taken an HIV test. Another survey was conducted in the general population among 621 adult inhabitants in Saint-Georges-de-l'Oyapock and Oiapoque between October 2017 and February 2018. This survey showed that over 20% reported high risk sexual activities in the past year. High HIV risk behavior included non-systematic use of condoms with a casual sex partner (*n* = 102, 16.4%), or commercial sex partners (*n* = 11, 1.8%) and/or having at least two sexual partners (*n* = 88, 14.0%). This overall context thus presents multiple opportunities for the spread of sexually transmitted infections.

Behavioral change and systematic condom use for casual sex is a substantial challenge. For HIV, diagnosing and treating effectively most patients as early as possible remains a major way to reduce HIV transmission. For hepatitis B and HPV, the available vaccines are still rarely used, although hepatitis B vaccine coverage may be increasing, HPV vaccination, which is expensive, remains exceptional. In such remote primary health care settings, bacterial STI case management and secondary prevention by screening or treatment to prevent complications are hampered by the absence of affordable and accessible diagnostic tests. For now, case management of STIs essentially rests on syndromic management. Such an approach has poor specificity, and leads to overprescription of antibiotics (38). Perhaps more importantly, The syndromic approach does not have a major impact on transmission because a very high proportion of infections are asymptomatic, and even then they may still cause inflammation leading to harmful sequelae. Partner notification in such a migration hub remains challenging but Non-Government Organizations may help to reach them.

The era of pure syndromic management may be coming to an end as rapid and simple point-of-care tests now seem to be promising solutions for more targeted STI case management and control (39, 40). Their fast turnaround times allows testing, communication of the results, treatment and follow-up plan, during the same consultation. All sexually transmitted infections do not have the same level of available tests: for HIV, HBV, Syphilis affordable, highly sensitive, and specific point-of-care tests are available. However, the available point-of-care tests for chlamydiasis and gonorrhea have had low accuracy or required expensive equipment. Recently, new tests were approved by the FDA (41) but their implementation in the context of the border between French Guiana and Brazil may not be feasible or affordable. Currently, for syphilis and trichomoniasis the point-of-care tests meet the ASSURED benchmark but this is not the case for chlamydiasis or gonorrhea. How these tools should be integrated in a shared strategic policy against sexually transmitted infections remains to be determined and a coordinated response between the Brazilian and French health professionals seems essential.

## Conclusions

Populations and patients on the border migrate and one could say the same about health professionals who mostly have short stays in these remote areas. The constant turnaround does not help follow-up; it also does not help capitalizing knowledge and grasping the big picture. Intuitive beliefs about HIV in this area can be wrong and there is a need for systematic data collection and exchange, and there is a need to synthesize and consolidate the available knowledge. This is still very much lacking. Although, from the limited data presented here, incidence of Chlamydiasis and gonorrhea seems lower that in the Americas, Syphilis does seem to be more frequent and warrants a special focus. The present review and the efforts to show the evolution of testing and care, and to estimate the size of the population of persons living with HIV may help the health professionals and NGOs to optimize their efforts to alleviate the burden of sexually transmitted infections. As we have shown above, the ongoing cooperation between multiple partners on both sides of the border has led to remarkable progress in primary prevention, in testing efforts, in treatment and retention on both sides of the border. Cooperation should be sustained because, in this area where sexual risk taking is common, interrupting the efforts to control STIs would rapidly translate in soaring transmission.

## Author contributions

MN: conception, analysis, and first and final draft writing. MGa and CM: investigation. SRa, CL, FD, and SRh: data curation. MGa, CM, MGo, PP, AA, AS, CR, CL, DM, VC, and AL: validation and review and editing. All authors contributed to the article and approved the submitted version.
