# Supplementary figures and images for: Sexually transmitted infections on the border between Brazil and French Guiana
Source: Front Public Health. 2023 Jan 25;11:1059137. doi: 10.3389/fpubh.2023.1059137 (PMC9906991; doi:10.3389/fpubh.2023.1059137)

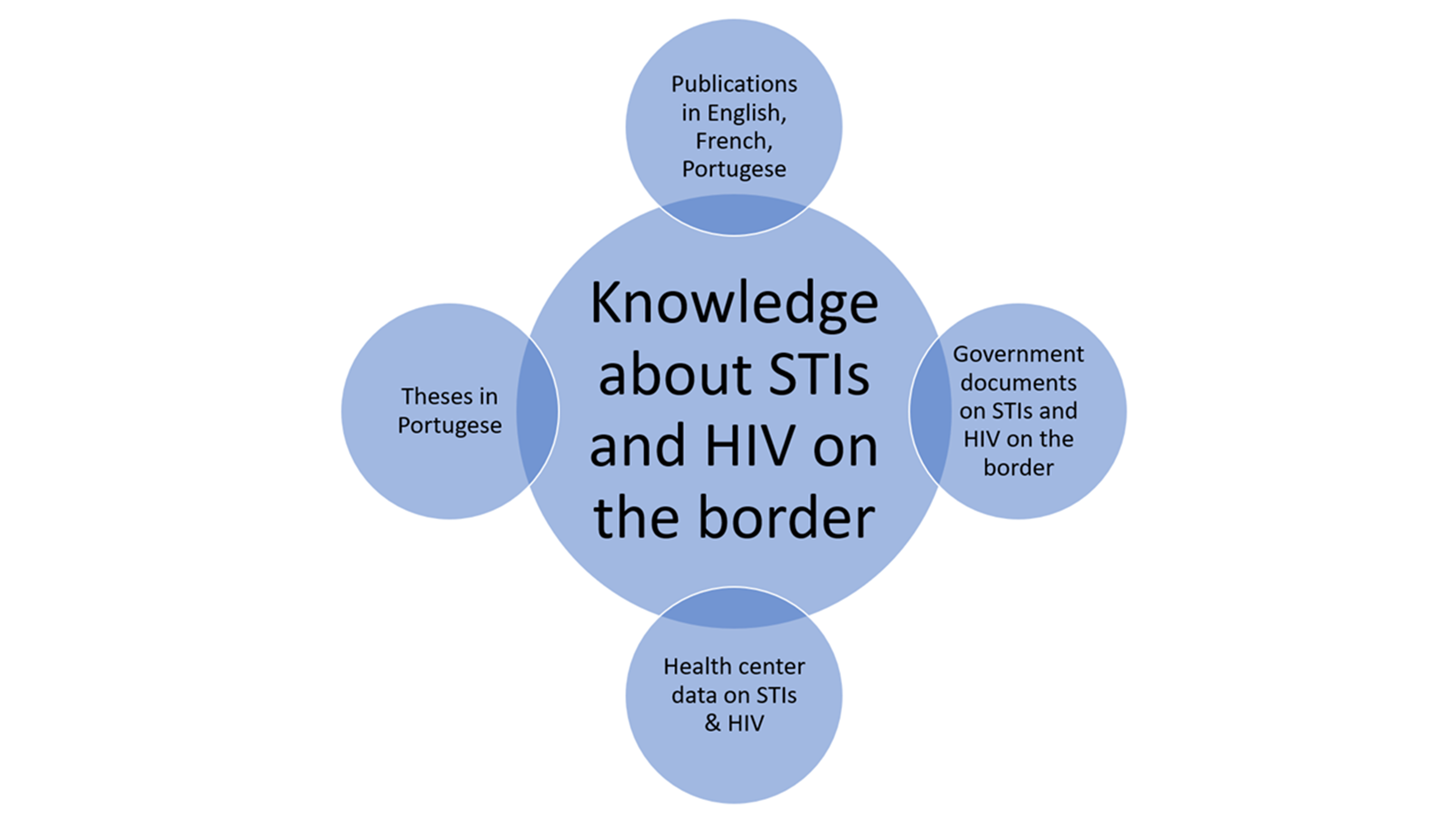

Supplement: Supplementary file 1 [file Image_1.TIF]

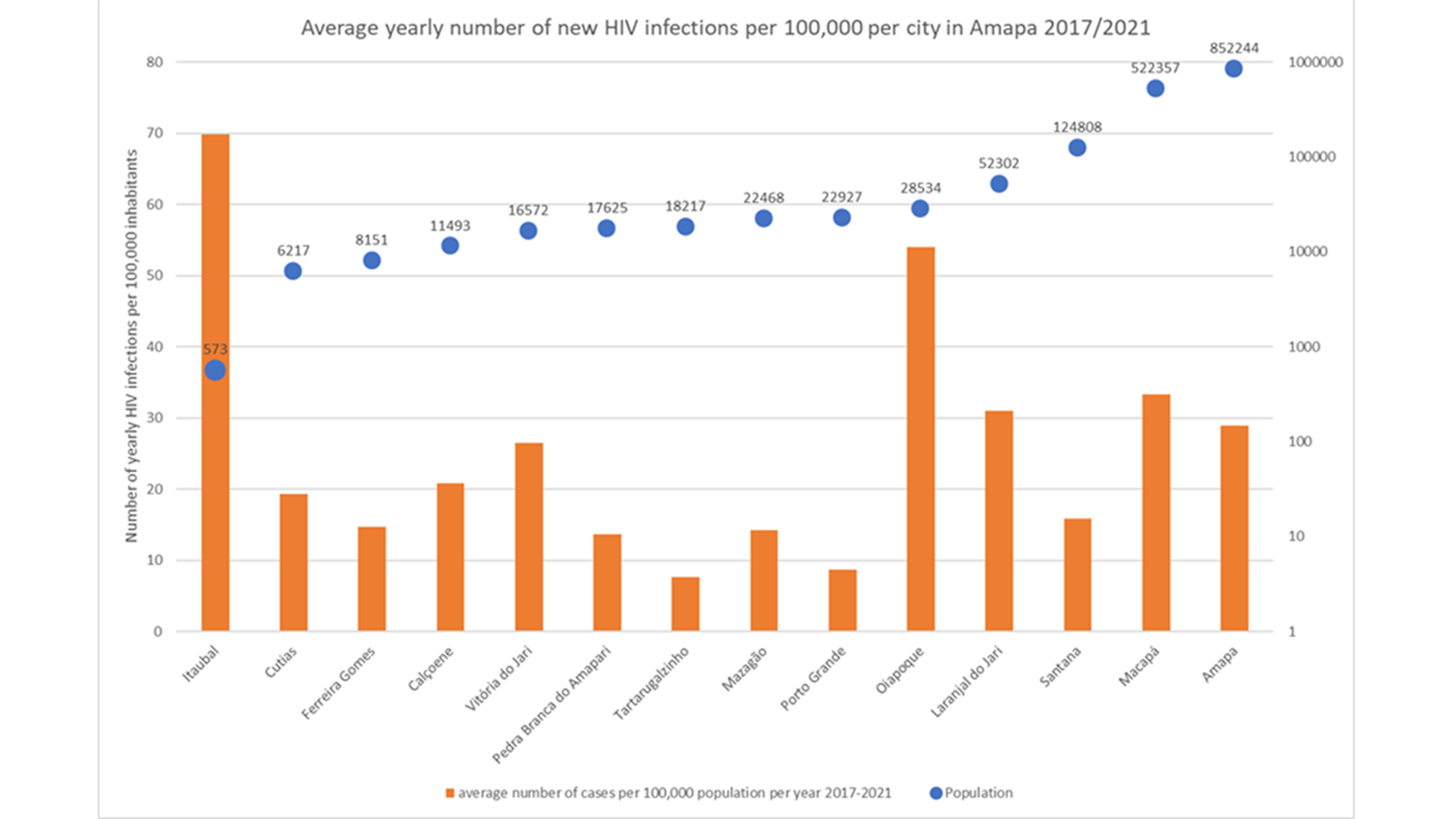

Supplement: Supplementary file 2 [file Image_2.TIF]

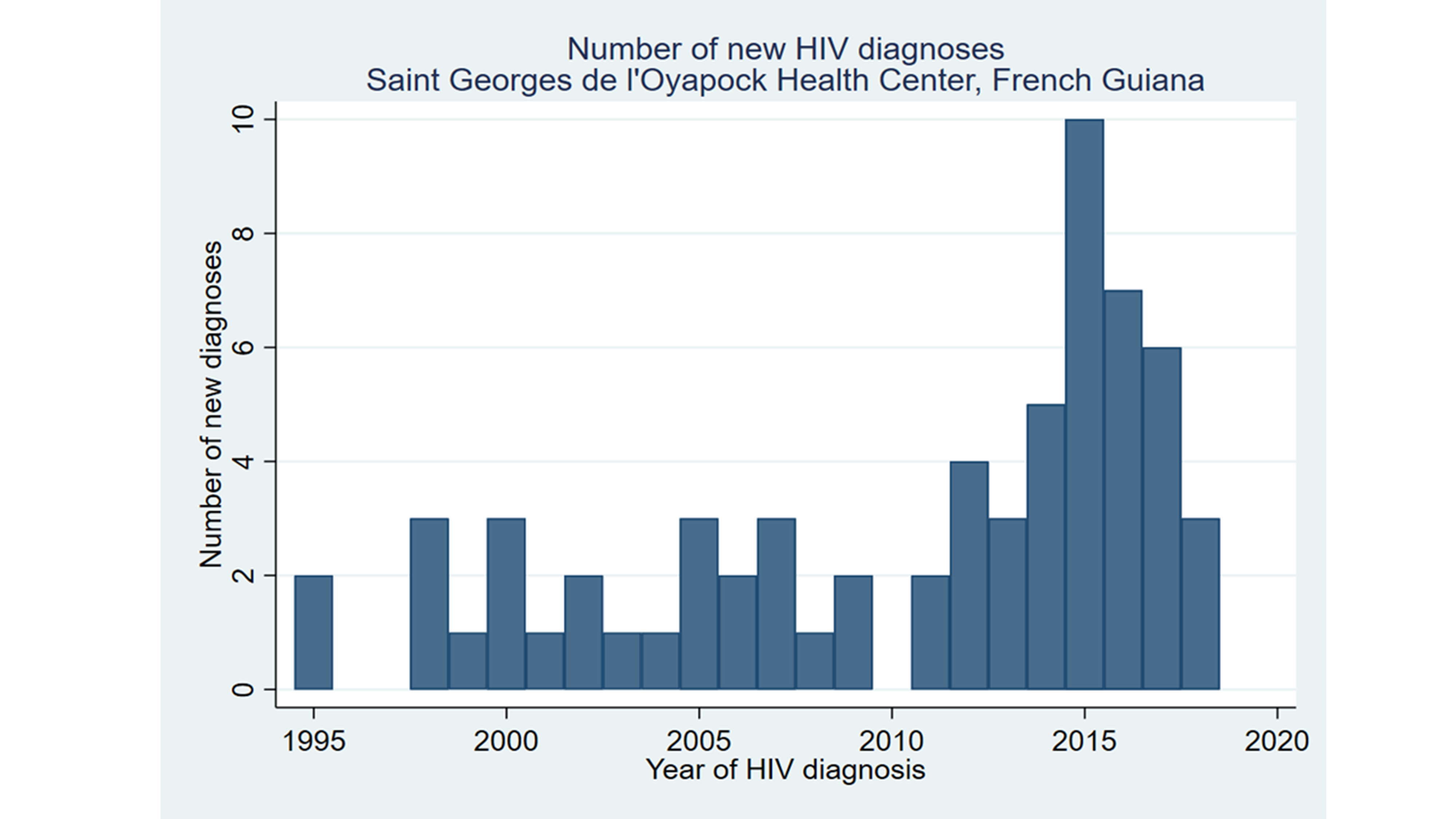

Supplement: Supplementary file 3 [file Image_3.TIF]

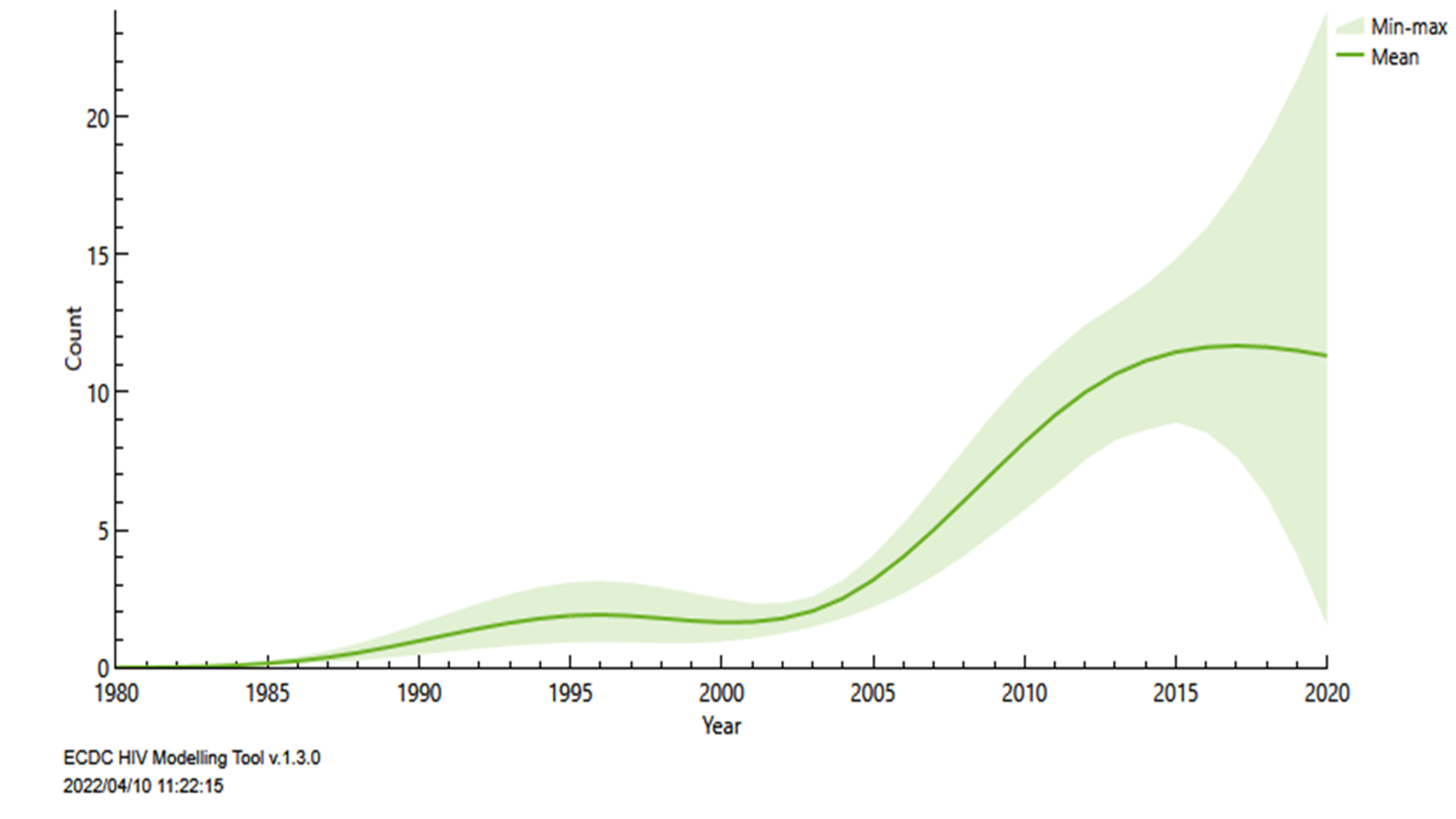

Supplement: Supplementary file 4 [file Image_4.TIF]

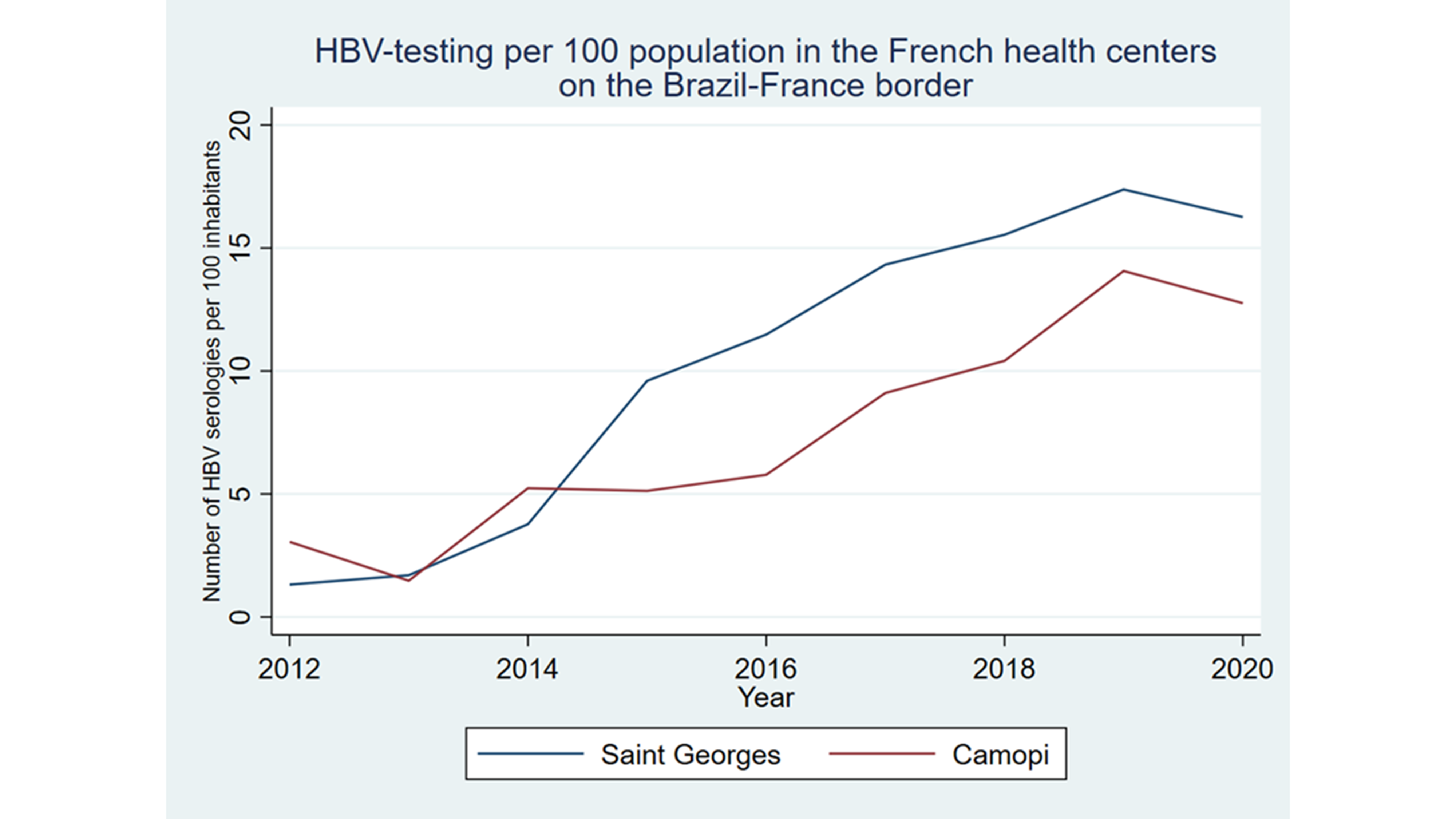

Supplement: Supplementary file 5 [file Image_5.TIF]

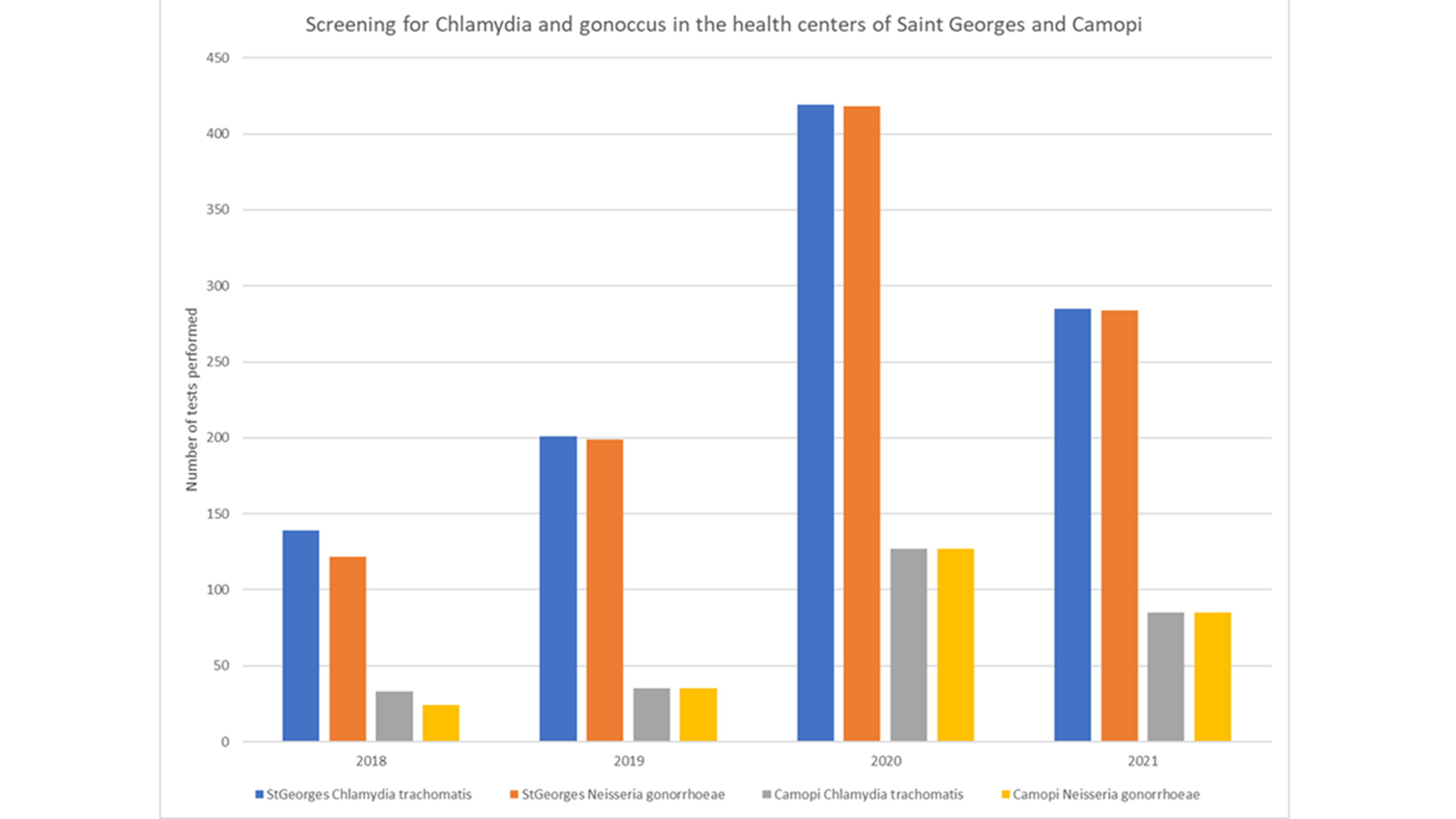

Supplement: Supplementary file 6 [file Image_6.TIF]

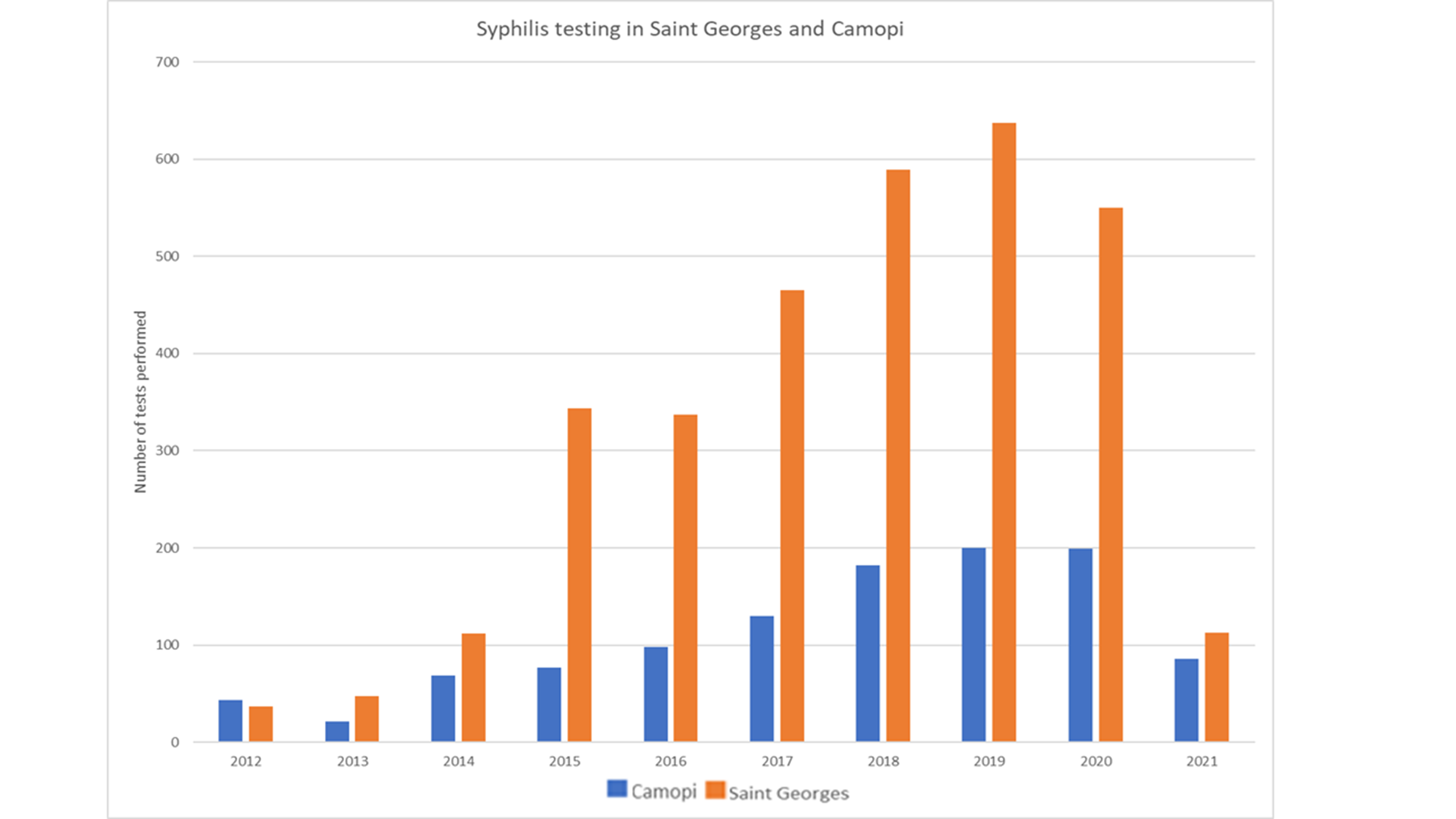

Supplement: Supplementary file 7 [file Image_7.TIF]
